# Hypermethylation of brain natriuretic peptide gene is associated with the risk of rheumatic heart disease

**DOI:** 10.1042/BSR20160408

**Published:** 2017-01-16

**Authors:** Ni Li, Dawei Zheng, Lebo Sun, Huoshun Shi, Xiuying Zhu, Guodong Xu, Qinning Wang, Caimin Zhu, Guofeng Shao

**Affiliations:** 1 Ningbo Medical Center Lihuili Hospital, Ningbo University School of Medicine, Ningbo, Zhejiang 315041, China; 2 Ningbo University School of Medicine, Ningbo, Zhejiang 315211, China

**Keywords:** DNA methylation, rheumatic heart disease, brain natriuretic peptide, pyrosequencing, epigenetics

## Abstract

To investigate the contribution of brain natriuretic peptide (BNP) promoter DNA methylation to the risk of rheumatic heart disease (RHD) and the influence of warfarin anticoagulant therapy on BNP methylation levels for RHD patients after surgery. BNP methylation levels were determined by bisulfite pyrosequencing from plasma samples of RHD patients compared with healthy controls. Several factors influencing the RHD patients were included like age, smoking and cholesterol levels. A fragment of five CG sites (CpG1–5) in the promoter region of *BNP* gene was measured. *BNP* gene hypermethylation was found in CpG4 and CpG5 in RHD patients compared with non-RHD controls. A significant difference was also observed between RHD patients with long-term administration of warfarin and RHD patients who had recently undergone an operation. Moreover, single CpG4 and CpG5 analysis revealed a significant increase in methylation levels in men. *BNP* gene body hypermethylation is associated with the risk of RHD, and also influenced by the warfarin anticoagulant therapy of RHD patients after surgery, which could represent novel and promising targets for therapeutic development.

## Introduction

Rheumatic heart disease (RHD) is primarily an autoimmune sequela of acute rheumatic fever, which occurs as a result of β-haemolytic streptococcal infection [1]. RHD can cause chronic inflammation of the endocardium and myocardium, leading to valvular dysfunction and haemodynamic changes and commonly heart failure, stroke or other serious-related complications [2,3]. Unfortunately, because of the lack of a specific method of detecting RHD, many patients have been diagnosed with irreversible valvular dysfunction, for which valvular surgery is one of the main treatments [4,5]. In China, RHD remains a significant health burden, although the prevalence of RHD has been declining in recent years. Owing to the lack of early detection, RHD patients can suffer from serious complications and irreversible valvular dysfunction. The identification of a biomarker of characteristic RHD pathophysiology will be valuable to aid early detection and enable patients to avoid surgery by starting effective treatment at an early stage.

DNA methylation often occurs in a CpG dinucleotide context and promoter DNA methylation can regulate the expression level of the gene [6,7]. Aberrant DNA methylation has been extensively studied in cancer, lung cancer and leukaemia [8–10]. It is often related to the regulation of gene expression [11,12]. DNA methylation plays a major role in rheumatic diseases [13]. However, only a few studies have indicated the involvement of DNA promoter methylation in the susceptibility of RHD and studies of the epigenetic mechanism in RHD are scarce.

Anticoagulation is a key treatment for patients with RHD after valve replacement, with the aim of prolonging the patient’s prothrombin time, preventing thrombosis in the artificial heart valve and preventing artificial valve dysfunction. Therefore, anticoagulation therapy is the principal method for follow-up treatment after valve replacement. Warfarin is a common anticoagulant drug in the clinic, and the inhibition of vitamin K epoxide reductase activity prevents vitamin K-dependent activation of clotting factors to exert an anticoagulant effect. Clinical practice shows that the safety of warfarin in the treatment of narrow scope and an improper dosage will lead to ineffective treatment or bleeding [14]. A large number of studies show that individual warfarin dosage fluctuations depend on differences in individual drug metabolism, including non-genetic and genetic factors [15–17]. It is unclear whether long-term warfarin anticoagulation therapy could influence the genetic-level impact including DNA for RHD patients undergoing mechanical heart valve replacement.

At present, several genes are reported to be associated with RHD pathogenesis including the brain natriuretic peptide (*BNP*), *BMPR2* and *GCK* genes [18–20]. BNP is a cardiac hormone synthesized as a pre-propeptide, mainly expressed in the ventricle, when left ventricular function is insufficient. It is also reported that BNP is a hallmark for maladaptive remodelling of the left ventricular function, because myocardial expansion and rapid synthetic released into the blood can help to regulate cardiac function [21,22]. *BNP* gene encoded by BNP consists of three exons and two introns, which is located in the short arm of chromosome 1, produced by the cardiomyocytes in the capacity of overloaded stress. *BNP* gene expressed in atrial myocytes and ventricular myocytes, mainly from ventricular myocytes especially in pathological state. It is first synthesized as pro-BNP hormone precursor in the myocardial cell, then removed by lysis of Ala^26^ amino acid signal peptide and finally secreted into the cell in the form of prepro-BNP. The biological half-life of BNP in circulatory blood is approximately 23 min. Many studies have proved that plasma BNP levels from blood are known to be elevated in patients with symptomatic left ventricular dysfunction, and plasma BNP concentrations can be a diagnostic performance of the heart disease marker. BNP acted mainly as a cardiac hormone and is produced predominantly in the heart that is involved in the regulation of natriuresis, diuresis and blood flow. *BNP* gene expression increases dramatically in response to hypertrophic stimuli, and plasma BNP levels are used clinically to detect and guide the management of hypertrophy and heart failure in humans [23–25]. Since BNP expression increases in response to elevated haemodynamic load, predicting clinical severity and prognosis in patients with heart failure, we analysed methylation patterns in the promoter regions by bisulfate pyrosequencing. *BNP* gene expression and its quantified expression locus might be associated with RHD. In light of previous findings, we hypothesized that promoter DNA methylation of *BNP* gene in peripheral blood might contribute to the risk of RHD. Thus, the goal of the present study was to assess whether *BNP* gene promoter DNA methylation is associated with the risk of RHD. Our work may identify novel DNA methylation markers and enhance current understanding of RHD progress after surgery.

## Materials and methods

### Patients

A total of 100 subjects were selected for the study from the inpatient clinic of Ningbo Medical Center, Lihuili Hospital (Ningbo, China) between March 2011 and October 2015. Of these subjects, 35 men and 35 women RHD patients (case group) and the remaining 30 were normal healthy adults (control group) with no medical history of congenital heart disease, cardiomyopathy, liver or renal diseases. The inclusion criteria of the RHD group were as follows: (i) every patient diagnosed with mitral valve prolapse because of mitral chordae tendinae fracture and mitral insufficiency and scheduled for mitral valve replacement; (ii) left ventricular ejection fraction (EF) >50%; (iii) left ventricular end-diastolic diameter (LVEDD) <55 mm. RHD cases and their controls were well matched based on the following details: (i) same sex; (ii) difference of age <5 years old; (iii) other physiological indexes from physical check. All human materials were obtained by the hospital’s regulations and hence were approved by the Ethics Committee of Lihuili Hospital, Ningbo, China. Written informed consent was also obtained from all subjects in advance.

### Sample collection

Blood samples were collected in EDTA tubes for plasma collection from 70 RHD cases. Peripheral blood was coagulated at room temperature for 30 min, then centrifuged at 3000 rpm for 15 min to completely remove cell debris and stored at −80°C. All plasma samples of cases and controls were collected by the same investigators.

### Bisulfate treatment

Bisulfate treatment was performed on 500 ng genomic DNA with the EpiTect^®^ DNA bisulfate kit (Qiagen, Germany) according to the manufacturer’s instructions.

### DNA methylation analysis pyrosequencing

PCR of bisulfite-treated DNA was amplified in a total volume of 38 μl PreMix, 50 pmol of each primer (forward primer: AGGATTAGTAGGTAGGTAGGGTGTATA and reverse primer: CCCCCTTCTTCCTTTCCTACAAATATCCAA) and 2 μl of bisulfite DNA. Forty cycles were carried out; the PCR conditions are shown in Table 1. PCR products were visualized on 1.5% agarose gels. Pyrosequencing was carried out using the sequencing primer GTAGGTAGGGTGTATAG and the PyroMark Q96 kit according to the manufacturer’s instructions on a PyroMark Q96 System (Qiagen). In total, five CpGs of BNP intron 1 were analysed in the pyrosequencing detector (PyroMark Q96 ID) using Pyro Q-CpG software.

**Table 1:** PCR primer concentrations and cycling conditions

	Primer forward (50 pM/μl)	Primer reverse (50 pM/μl)	Cycling conditions
BNP	1 μl	1 μl	95°C for 3 min
			94°C for 30 s, 58°C for 30 s, 72°C for 1 min; for 40 cycles
			72°C for 7 min

### Data evaluation 

For pyrosequencing, DNA quality was controlled using BiQ Analyzer software [33]. Data are shown as means ± S.D. SPSS version 22 was used for statistical evaluation; *P*<0.05 was regarded as significant. Student’s *t* test was used to test for differences in methylation levels for categorical variables, Mann–Whitney test was used by checking three option boxes for the influence of warfarin.

## Results

### DNA methylation levels of BNP

**Figure 1:**
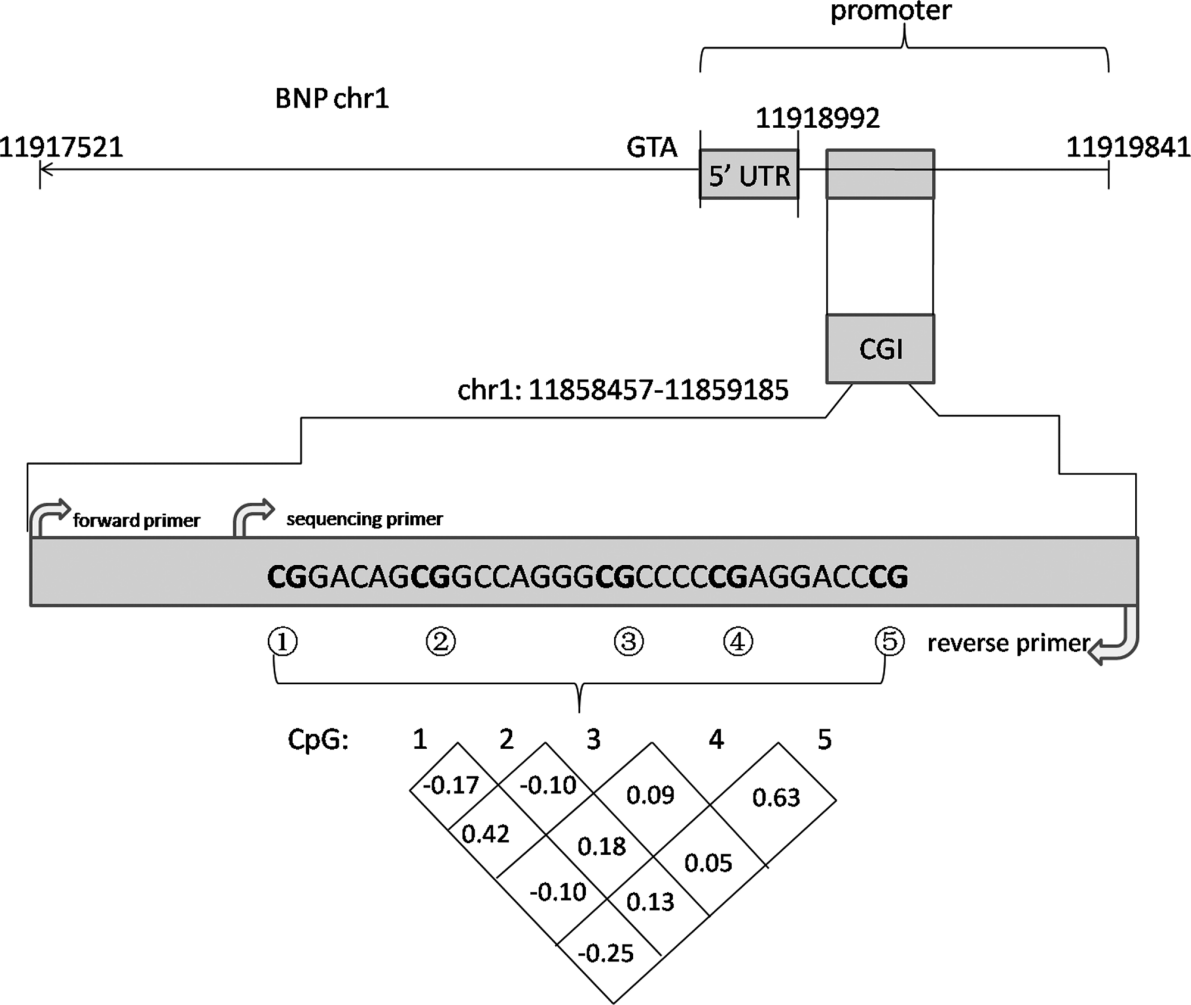
Primer information and correlation coefficient among the five CpGs in *BNP* gene promoter

**Figure 2:**
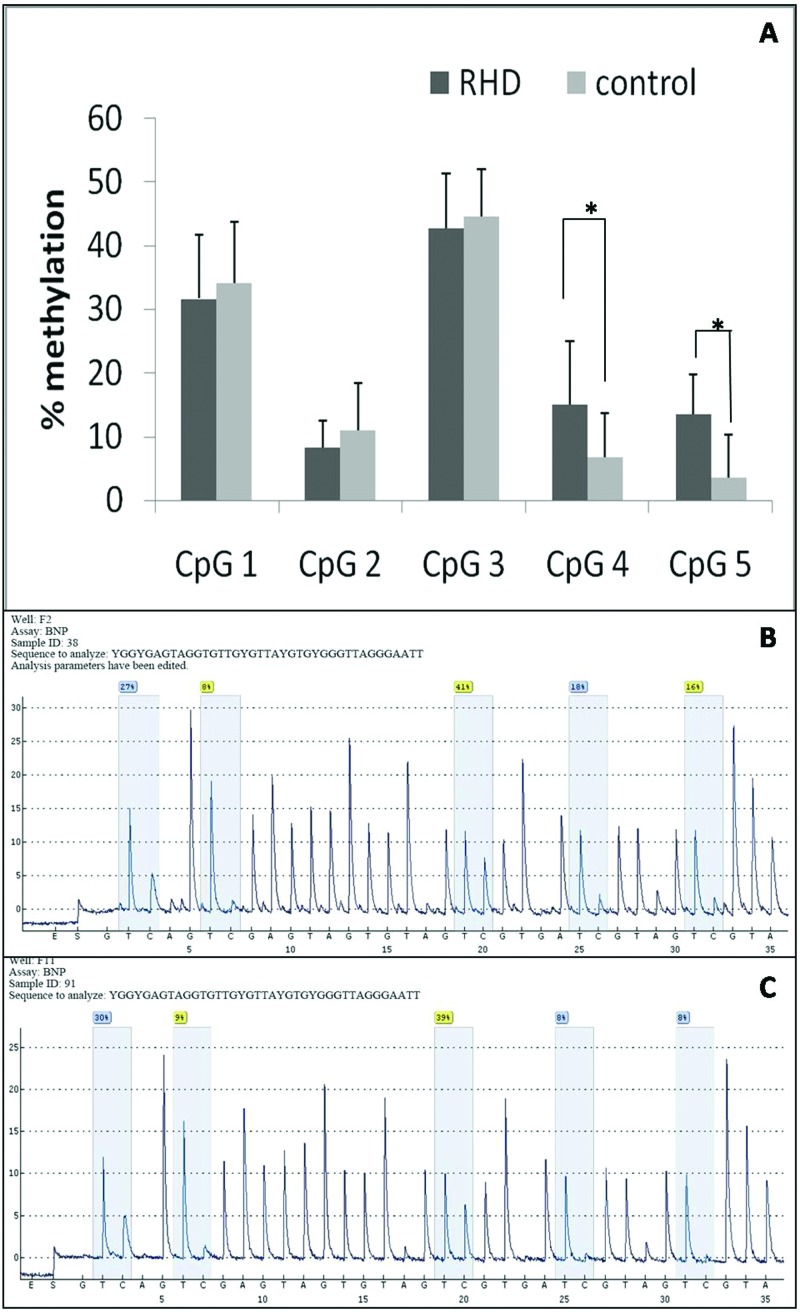
Methylation levels (%) of BNP-specific methylation markers at five different CGIs (**A**) The methylation levels of five CpG sites on *BNP* gene body of one RHD sample. (**B**) The methylation levels of five CpG sites on *BNP* gene body of one control sample (*P<0.05). (**C**) The methylation levels of five CpG sites on *BNP* gene body in all RHD groups and healthy controls.

As shown in Figure 1, the bisulfite pyrosequencing assay was carried on a fragment in the promoter region of the *BNP* gene. This fragment contained five CG sites that could be measured to evaluate the methylation levels of the *BNP* gene promoter. DNA methylation of the *BNP* gene in the RHD cases was significantly higher than in the non-RHD controls. Correlation coefficient of the DNA methylation levels was measured among these five CpGs. To determine the DNA methylation level of RHD, we chose five CpG islands (CGI) of RHD and controls. We found significant differences at CpG4 and CpG5 using pyrosequencing (shown in Figure 2).

### Warfarin therapy for DNA methylation levels of BNP

**Figure 3:**
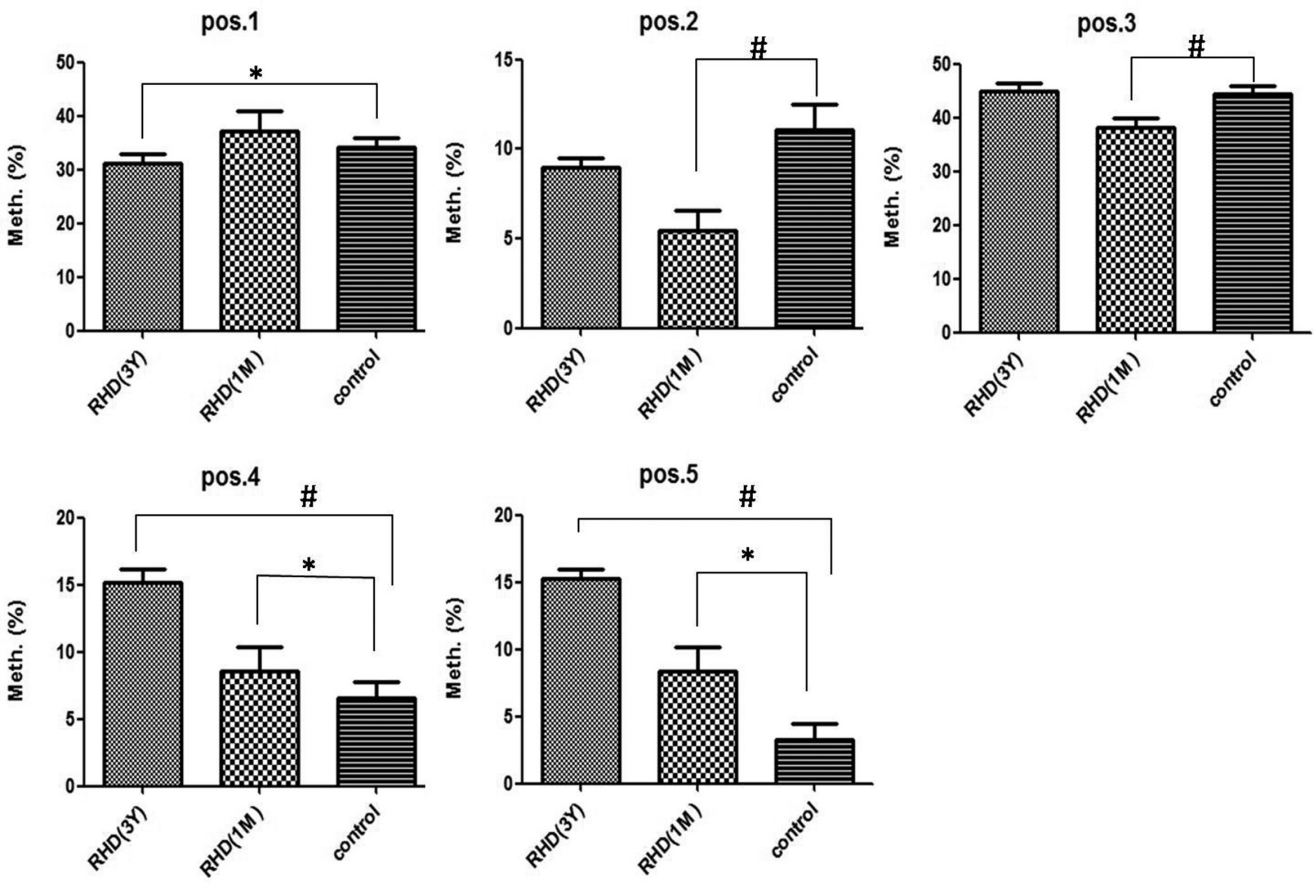
The influence of warfarin anticoagulant haemorrhage with different times on BNP DNA methylation levels (pos.1, pos.2, pos.3, pos.4 and pos.5 are five different CG sites) (^#^*P*<0.01,*P<0.05)

We next investigated the effect of BNP DNA methylation levels on warfarin dose requirements after valve replacement. As shown in Figure 3, we found a significant difference on the CpG4 (pos.4) site and CpG5 (pos.5) site, in comparison with 1 month (RHD (1M)), 3 years (RHD (3Y)) and the control group. With sequencing methods, the higher methylation levels were detected at CpG4 and CpG5 after 3Y of taking warfarin compared with 1M. It also analysed the data of CpG2 (pos.2), the difference between 1M and control group is very significant (*P*=0.0033), and the significance of CpG3 (pos.3) between 1M and control group was also found (*P*=0.0045), the differences of other groups were not significant, the significance of CpG1 (pos.1) between 3Y and control group was also found (*P*=0.0335) (shown in Figure 3).These results indicated that long-term warfarin anticoagulation therapy could influence the genetic-level impact including DNA for RHD patients undergoing mechanical heart valve replacement.

### DNA methylation levels of BNP in men and women

**Figure 4:**
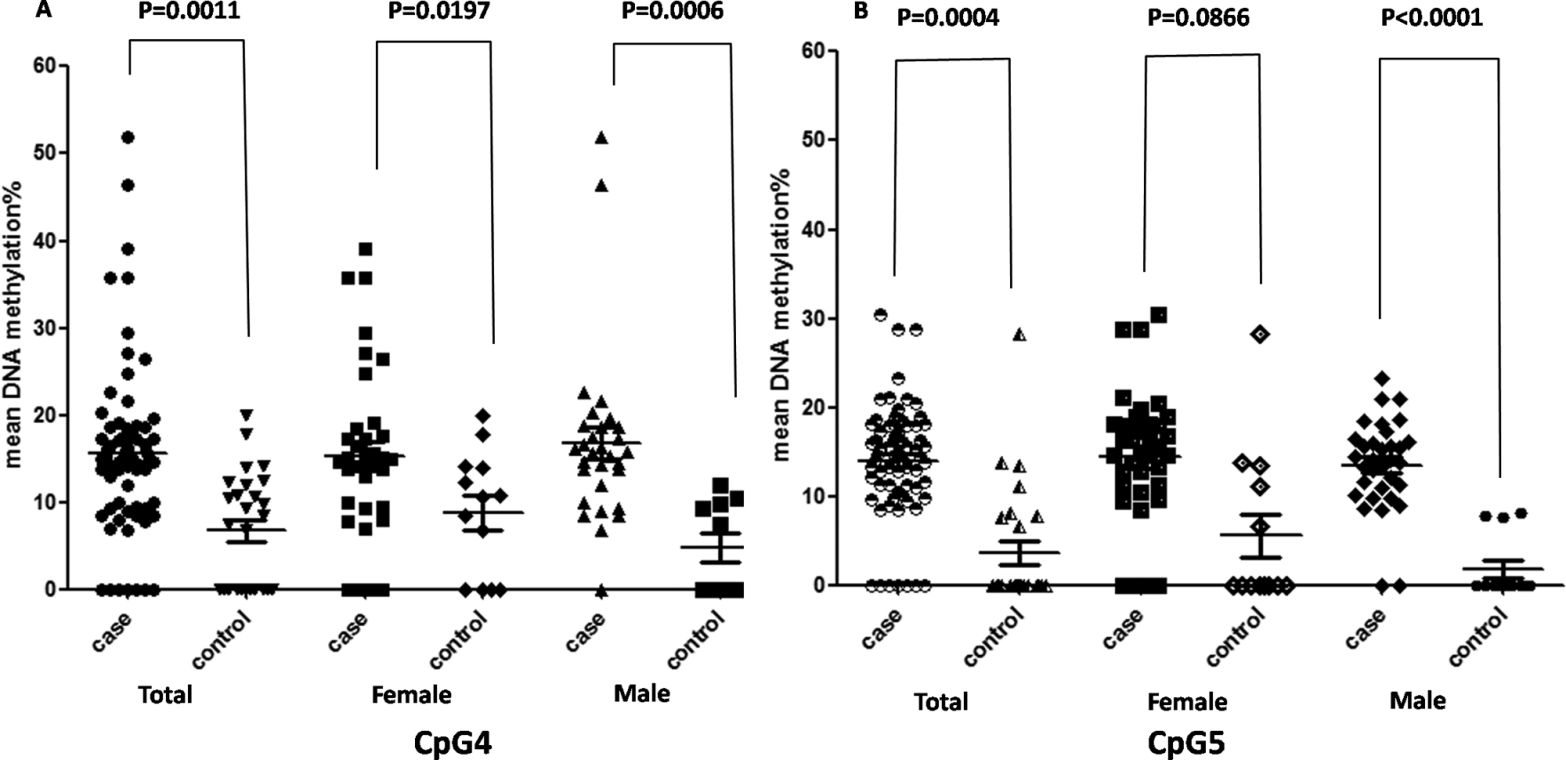
Comparison of BNP methylation levels according to sex between cases and controls; (A) CpG4 and (B) CpG5

We compared the methylation status of pos.4 and pos.5, in order to determine whether DNA methylation levels of BNP CpGs show significant difference between men and women. We could not detect a significant difference between men and women (Figure 4). However, single CpG analysis revealed increased significant methylation levels in men at different sites including CpG4 and CpG5, whereas in women the difference was not significant (Figure 4).

As shown in Table 2, a total of nine phenotypes were involved in the present study. Among these phenotypes, there were significant differences between men and women on phenotypes including serum total cholesterol (TC), triacylglycerol (TG) and apolipoprotein B (ApoB). No significant association of rest of the phenotypes with the risk of RHD was found (Table 2).

**Table 2:** Characteristics of subjects according to sex

Characteristics	Men (*n*=35); mean ± S.E.M.	Women (*n*=35); mean ± S.E.M.	*P* value
Age	57.43 ± 12.25	52.82 ± 13.06	0.15
TG (mmol/l)	1.19 ± 0.52	1.42 ± 0.35	0.19
TC (mmol/l)	4.16 ± 1.31	5.04 ± 0.63	0.02
HDL (mmol/l)	1.12 ± 0.23	1.25 ± 0.14	0.05
LDL (mmol/l)	2.54 ± 0.96	3.15 ± 0.62	0.02
ApoA (g/l)	1.07 ± 0.23	1.16 ± 0.27	0.17
ApoB (g/l)	0.86 ± 0.24	1.03 ± 0.18	0.03
ApoE (g/l)	4.18 ± 1.08	5.28 ± 1.89	0.14
Smoke or not	4/35	0/35	/
CpG1	31.15 ± 10.40	32.23 ± 9.87	0.58
CpG2	7.87 ± 3.14	8.74 ± 5.04	0.32
CpG3	42.27 ± 8.87	43.15 ± 8.32	0.63
CpG4	15.43 ± 10.14	14.56 ± 8.32	0.72
CpG5	13.29 ± 4.90	14.56 ± 9.73	0.41
Mean BNP methylation (%)	21.02 ± 4.65	22.08 ± 4.68	0.96

Abbreviations: ApoA, apolipoprotein A; ApoE, apolipoprotein E; HDL, high-density lipoprotein; LDL, low-density lipoprotein.

### DNA methylation levels of BNP in RHD with different ages

**Figure 5:**
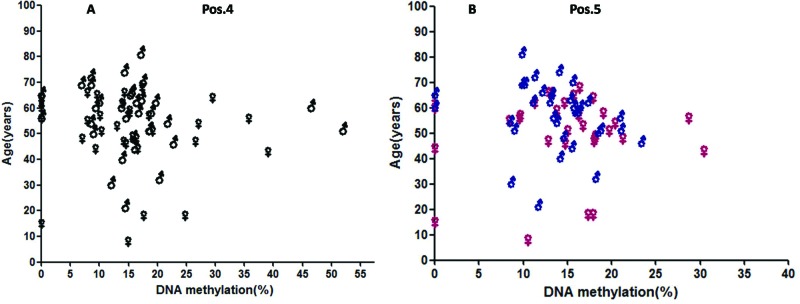
Correlation between BNP methylation and age; (A) CpG4: pos.4 and (B) CpG5: pos.5

Age can also be a factor that influenced DNA methylation level in cardiac disease. We detected elevated DNA methylation levels in different ages of RHD patients. However, the difference was not significant, although lower methylation levels were found in older cases (shown in Figure 5).

## Discussion

Epigenetic modifications, such as DNA methylation and histone modifications, represent attractive disease mechanisms because they might help to explain how environmental and lifestyle factors can impose aberrant gene expression patterns in an individual’s lifetime that can result in increased cardiovascular risk [19,26–28]. RHD cases were observed to have significantly higher genomic DNA methylation in peripheral blood in comparison with non-RHD controls. In addition, candidate epigenetic analysis showed that altered BNP promoter DNA methylation was associated with the risk of cardiovascular disease. In the present study, we recruited RHD cases and healthy controls to test the association between DNA methylation of the *BNP* gene and the risk of RHD. Currently, treating RHD usually involves conservative treatment and interventional therapy, while surgical treatment is considered to be the most effective method. Anticoagulation is a key treatment for patients with RHD after valve replacement, with the aim of prolonging the patient’s prothrombin time, preventing thrombosis in the artificial heart valve and preventing artificial valve dysfunction [29,30]. Therefore warfarin, which is considered a common anticoagulation drug with long-term therapy, may influence the genetic-level impact including DNA for RHD patients undergoing mechanical heart valve replacement. Herein, we studied the cardiovascular-specific gene *BNP* DNA methylation levels to analyse their epigenetic factors.

We found significantly higher DNA methylation levels of the *BNP* gene in two CG sites, namely CpG4 (pos.4) and CpG5 (pos.5) in the RHD cases than in the non-RHD controls. This indicated that *BNP* gene body hypermethylation is associated with the risk of RHD.

A significant difference was also observed between RHD cases with a long-term dose of warfarin and RHD cases those who had recently undergone an operation. Clinical practice shows that the safety of warfarin in the treatment of narrow scope, and an improper dosage will lead to ineffective treatment or bleeding. Fluctuations of individual warfarin dosages can influence the severity of RHD in genetic factors. At present, researches showed usage of warfarin on stable doses on RHD causing *CYP2C9*, *CYP4F2* and *VKORC1* gene polymorphisms in the Chinese Han population [31,32]. In our study, the long-term anticoagulant therapy of warfarin in RHD patients after valve replacement changed expression levels of BNP DNA methylation sites in the field of apparent genetics. However, study of the mechanism of the BNP DNA methylation pattern changes in RHD is ongoing, by regulating *BNP* gene transcription, imprinting and defence invasion of exogenous genetic material in cardiac cells.

Single CpG analysis revealed increased significant methylation levels in men at different sites including pos.4 and pos.5, whereas in women the difference was not significant. Meanwhile, lower methylation levels were found in older cases. We thus hypothesized that age and sex-related alterations of BNP methylation might underlie the increased susceptibility towards RHD. The present study sought to determine (i) whether alterations of BNP intron methylation occurred during aging, (ii) whether the methylation pattern differed between men and women and (iii) whether cardiac cells exhibited different methylation patterns compared with non-cardiac cells. However, the above hypothesis should be further studied.

There were some limitations to our study. First, the sample size was relatively limited. Future investigation with more samples will need to be performed to confirm our findings. Second, only a fragment of the CGI was selected to stand for the whole promoter of BNP. Third, although we attempted to control the confounding factors that may affect the methylation level of BNP, the possibility remained of an unknown factor that might confound the alteration of BNP methylation, for example any other drug effects for the studied RHD patients. Fourth, we did not explore why BNP methylation correlated with TC, TG and ApoB. The exact interactions among these remain to be explained in future work.

Epigenetic blood-based biomarkers might provide insights into the RHD process after surgery. We will further explore the relationship between methylation status and RHD development as well as clinicopathological prognostic factors. Further studying the DNA methylation of genes associated with RHD as BNP for early detection and diagnosis of RHD may help us determine the genesis and development of these mechanisms and devise better treatment for this disease.

## Conclusion

In conclusion, our results suggest *BNP* gene body hypermethylation is associated with the risk of RHD, and have influence on the warfarin anticoagulant therapy of the RHD patients after surgery, which could represent novel and promising targets for therapeutic development.

## Abbreviations

ApoA: apolipoprotein A
ApoB: apolipoprotein B
ApoE: apolipoprotein E
BMPR2: bone morphogenetic protein receptor type 2
BNP: brain natriuretic peptide
CG: cytosine guanine
CGI: CpG island
CpG: cytosine-phosphate-guanine
CYP2C9: cytochrome P450 2C9
CYP4F2: cytochrome P450 4F2
GCK: glucokinase
HDL: high-density lipoprotein
LDL: low-density lipoprotein
pos.: position
RHD: rheumatic heart disease
TC: serum total cholesterol
TG: triacylglycerol
VKORC1: vitamin K epOxide reductase complex subunit 1
1 M: 1 month
3 Y: 3 years
